# Self-Powered Non-Contact Triboelectric Rotation Sensor with Interdigitated Film

**DOI:** 10.3390/s20174947

**Published:** 2020-09-01

**Authors:** Zhihua Wang, Fengduo Zhang, Tao Yao, Na Li, Xia Li, Jianfeng Shang

**Affiliations:** 1 State Key Laboratory of Reliability and Intelligence of Electrical Equipment, Hebei University of Technology, Tianjin 300130, China; fenda_zhang123@163.com (F.Z.); jintian011@hebut.edu.cn (X.L.); sjf@hebut.edu.cn (J.S.); 2 Key Laboratory of Electromagnetic Field and Electrical Apparatus Reliability of Hebei Province, Hebei University of Technology, Tianjin 300130, China; 3 School of Mechanical Engineering, Hebei University of Technology, Tianjin 300130, China; yaotao@hebut.edu.cn; 4 Industrial Technology Center of Chengde Petroleum College, Chengde 067000, China; cdpc_ln@cdpc.edu.cn

**Keywords:** self-powered, triboelectric nanogenerators, rotation sensor, motion monitoring

## Abstract

Rotation detection is widely applied in industries. The current commonly used rotation detection system adopts a split structure, which requires stringent installation requirements and is difficult to miniaturize. This paper proposes a single-piece self-powered non-contact sensor with an interdigital sensitive layer to detect the rotation of objects. The electric field generated between a polyurethane (PU) film and a polytetrafluoroethylene (PTFE) film is utilized for perceiving the rotation. The surface of the PU film is subjected to wet etching with sulfuric acid to increase the surface area and charge density. Through finite element analysis and experimental testing, the effects of the areas of the sensitive films as well as the horizontal and vertical distances between them on the output voltage are analyzed. Tests are performed on adjustable-speed motors, human arms, and robotic arms. The results show that the sensor can detect the speed, the transient process of rotation, and the swing angle. The proposed rotation sensor has broad application prospects in the fields of mechanical automation, robotics, and Internet of Things (IoT).

## 1. Introduction

A rotational speed sensor is widely employed in modern industrial production, and is a core component for improving rotation control accuracy. Commercial speed sensors are mainly based on the principle of photoelectric effect or electromagnetic induction [1]. The sensor system is divided into two parts: a fixed part and a rotating part. The sensor requires a connector and a fixed bracket to fix the system on the ground or on an operating table. The installation is troublesome and is not conducive to the miniaturization and integration of rotation detection systems. In addition, with the rapid development of Internet of Things (IoT) and artificial intelligence technologies, rotational speed sensors will have broad applications. Scholars and enterprises have been focusing on developing new sensors characterized by low cost, ease of manufacturing, and reliable operation [2,3,4,5].

According to Maxwell’s displacement current theory, triboelectric nanogenerators (TENGs) can be fabricated by utilizing the triboelectric effect and electrostatic induction [6]. Polymers are widely used in manufacture of TENGs for its low cost and triboelectrification ability [7,8,9]. The manufactured structure is cost-effective and easy to process, making it ideal for research on micro/nano power supply and self-powered sensing [10,11,12,13,14,15,16,17,18,19]. Several scholars have studied self-powered rotational speed sensors. The earliest self-powered rotational speed sensors worked by periodic contact-separation between two different polymer films in direct contact. For example, a linear and rotary motion sensors based on the spiral arrangement of Kapton–copper tape can detect speeds in the range of 300–700 RPM with an error of less than 0.9% [20]. A disk TENG for collecting rotational mechanical energy and detecting speed is proposed [21]. However, sensors based on the direct friction of the sensitive layers have poor long-term stability because of the friction loss and heat generation under high-speed rotation. In order to solve this problem, sensors using rolling friction instead of sliding friction is proposed to reduce the friction material loss in high-speed rotation. A rotation sensor with a fully enclosed bearing structure can detect speeds in the range of 200–2200 RPM with an error of less than 0.09%, based on contact electrification between fine-sized rolling beads and a pair of planar electrodes [22]. A cylindrical multi-functional sensor can sense the direction, speed, and acceleration of linear and rotational motions simultaneously [23]. The results show that rolling friction can prolong the life of self-powered rotational speed sensor, but the output of TENGs will decrease and eventually fail under long-term operation [24,25,26]. In order to realize the long-time operation of rotating TENGs, researchers have designed non-contact TENGs. A disc-type non-contact rotating TENGs can realize non-contact rotational energy collection [27]. An automatic transition between contact and non-contact working states was demonstrated, which largely improved the robustness of the rotation system [28]. A non-contact clock-hand type self-powered counter has been used for wind speed detection [29]. It is considered to be a successful attempt of non-contact TENG as a rotational speed sensor. However, the stator and rotor need to be installed separately. This reduces its portability. Although the results show that non-contact TENGs have excellent long-term stability, the existing TENG-based rotation sensors are still similar in structure to the commercial ones and only suitable for specific scenarios, which is not conducive to their wide application in the Internet of things.

To overcome the shortcomings of ordinary rotational speed sensors that require a split structure as well as to avoid the effects of material loss on the output performance of TENG-based sensors, this paper proposes a self-powered non-contact triboelectric rotation sensor (SNTRS) with an interdigital film. First, the stator and rotor structures are connected by bearings, omitting the parts that need to be fixed on the ground or on the operating table, and then simply fixing the sensor onto the rotating shaft or object. Second, the non-contact design between the films can help effectively avoid the loss of sensitive materials and improve the service life and reliability of the sensor. Third, the interdigital film is designed with the objective to increase the measurement accuracy of the sensor for rotational speed and swing motion, and to distinguish the direction of rotation as well as identify transient processes. Fourth, a prototype is made and tested. The test results confirm the effectiveness of the SNTRS for rotation and swing motion sensing.

## 2. Methods

### 2.1. Design and Fabrication of a Self-Powered Rotation Sensor

**Figure 1:**
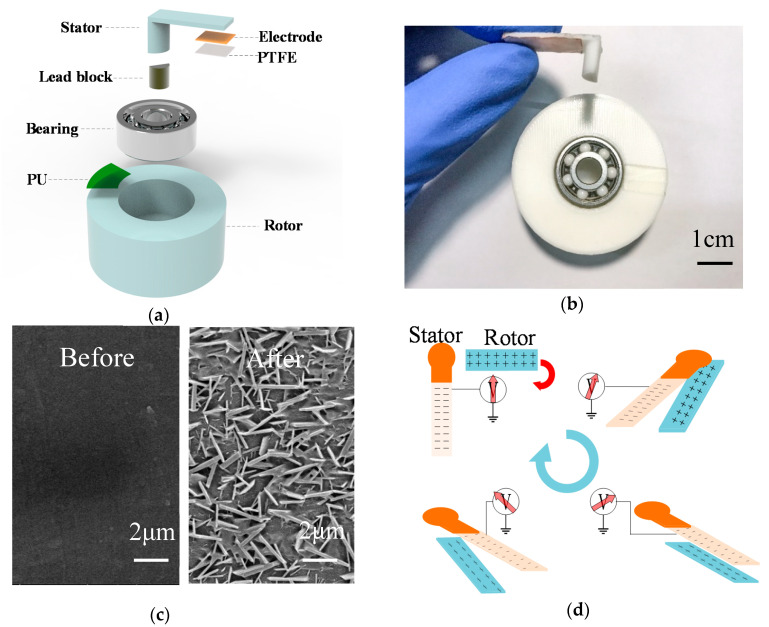
(**a**) Structure of the self-powered non-contact triboelectric rotation sensor (SNTRS), (**b**) Image of the SNTRS, (**c**) microstructure of the polyurethane (PU) film before and after wet etching with sulfuric acid, and (**d**) principle the sensor.

Figure 1a shows the 3D structure of a self-powered rotation sensor. Figure 1b shows the structure of the SNTRS comprising three parts: a stator part, a rotor part, and a contact ball bearing. The stator part is an L-shaped structure, with one end inserted into the inner shaft held by the bearing. The inserted part is embedded with a lead block to increase weight, so that the stator and the inner shaft remain relatively stationary with respect to the ground during rotation under the effect of gravity. The rotor part is a hollow cylindrical structure. The inner wall of the cylinder fits closely with the outer ring of the bearing. In the process of rotation speed detection, the rotor is fixed onto the measured rotating shaft or object. The outer shaft is driven to rotate along with the rotor. The polytetrafluoroethylene (PTFE) and polyurethane (PU) have high negative and positive charge affinities, respectively [30,31]. This makes the PTFE and PU ideal triboelectric materials. A PU film and a PTFE film with a thickness of 10 μm are pasted on the rotor and the stator, respectively. Copper electrodes are pasted on the back of the PTFE film for signal collection. The sensor can be installed only vertically, i.e., the center axis of the bearing is horizontal to the ground.

To improve the charge storage ability of the film, the PU film is immersed in a 3 mol L^−1^ dilute sulfuric acid solution for 2 min and then rinsed with water [30]. The surface of the treated PU film exhibits uneven micro/nanopatterns, which help in effectively increasing the charge level of the PU film so as to increase the output voltage. The surface morphology of the etched PU film is scanned using a Nova Nano SEM450 (FEI, Hillsboro, Oregon, USA) field-emission scanning electron microscope, as shown in Figure 1c.

### 2.2. Experimental Set-Up

The surface morphology of the etched PU film is scanned using a Nova Nano SEM450(FEI, Hillsboro, Oregon, USA) field emission electron microscope. A programmable motor with a maximum speed of 2200 RPM is used to produce a rotation at a steady speed. The DH8303 acquisition card (DC-100 kHz, 8-channel synchronous sampling) is used to synchronously acquire voltage signals. The KUKA Kr-6-R700 robot (six axis, A5 = ±120°, accuracy = 0.03 mm) is used in the swing experiment.

### 2.3. Sensing Mechanism

Figure 1d shows the working mechanism of the SNTRS. The surfaces of the pre-rubbed PU film and PTFE film have positive and negative charges, respectively. In addition, because the resistivities of PU and PTFE are relatively high, the charges accumulated on the film can be maintained for a long time.

When the rotor rotates, owing to the good lubricity of the bearing as well as the large weight of the lead block, the stator is always vertical to the ground under the action of gravity. The PU film on the rotor and the PTFE film on the stator undergo a periodic process in that they approach each other and then move away. When the PU film with positive charges approaches the PTFE film, the potential on the electrode above the PTFE film increases instantaneously, and a positive voltage peak appears. Meanwhile, the interfacial potential difference drives the positive charges to accumulate on the copper electrode. Subsequently, the PU film moves away from the PTFE film, and the copper electrode potential drops instantaneously. A negative voltage peak appears, while the interfacial potential difference drives the positive charges away from the copper electrode until a new electrostatic balance is attained.

## 3. Results and Discussion

**Figure 2:**
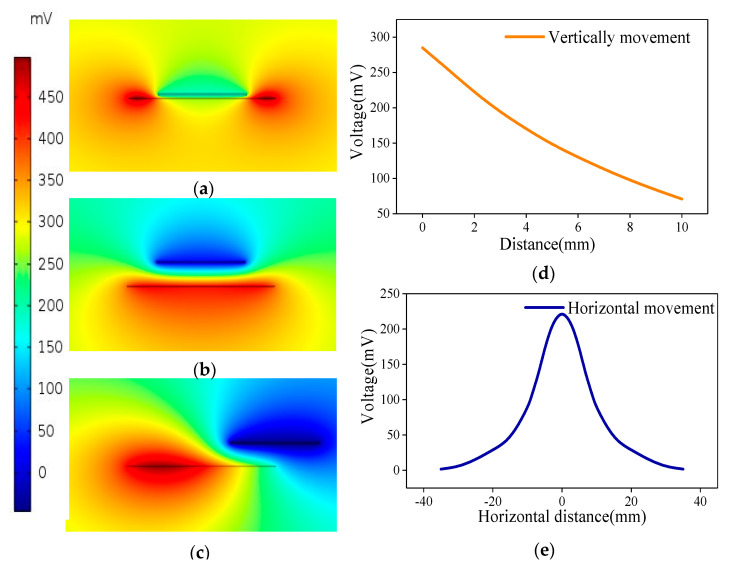
The electric potential distribution when the vertical distance between PU film and PTFE film is 0.1 mm (**a**) and 2 mm (**b**), (**c**) the electric potential distribution when the vertical distance between PU film and PTFE film is 2 mm and horizontal sliding, the relationship between vertical (**d**) or horizontal (**e**) distance and output voltage.

A finite element analysis was performed to calculate the electric field distribution and output voltage of the sensor. The size and materials of the simulation model are consistent with the parameters of the sensor prototype. The area charge density on the surface of the PU film and PTFE film are set to 240 and −400 pC m^−2^, separately. This is based on the principle of charge conservation and their respective areas. The positive charge on PU surface induces a positive electric field, while the negative charge on PTFE surface induces a negative electric field. The superposition of them forms the electrostatic field as shown in Figure 2a–c. A comparison between Figure 2a,b shows the effect of the vertical distance between the PU and PTFE on the potential distribution. The potential near the PTFE decreases with the increase in the distance between the PU and PTFE. A comparison between Figure 2b,c shows the impact of the horizontal distance between the PU and PTFE on the nearby potential when the vertical distance is fixed at 2 mm. As shown in Figure 2d, when the distance between the films is 0 mm, the potential at the copper electrode is 285 mV, and when the distance is 10 mm, the potential at the copper electrode is 70.8 mV. Figure 2e shows that when the PTFE is at the center position directly above the PU, the potential on the copper electrode is 222 mV. As the horizontal distance between the PTFE and PU increases, the potential on the copper electrode gradually decreases. When the horizontal distance increases to 35 mm, the potential on the copper electrode decreases to 1.8 mV. The finite element analysis result proves the feasibility of the SNTRS for non-contact sensing.

Clearly, the SNTRS can achieve non-contact rotation speed sensing. Moreover, the results prove that the potential on the copper electrode changes periodically with the periodic movement of the PTFE film on the stator and the PU film on the rotor. To verify the simulation results and quantify the relationship between the output voltage and the film width and that between the film distance and the velocity, the following tests were performed.

**Figure 3:**
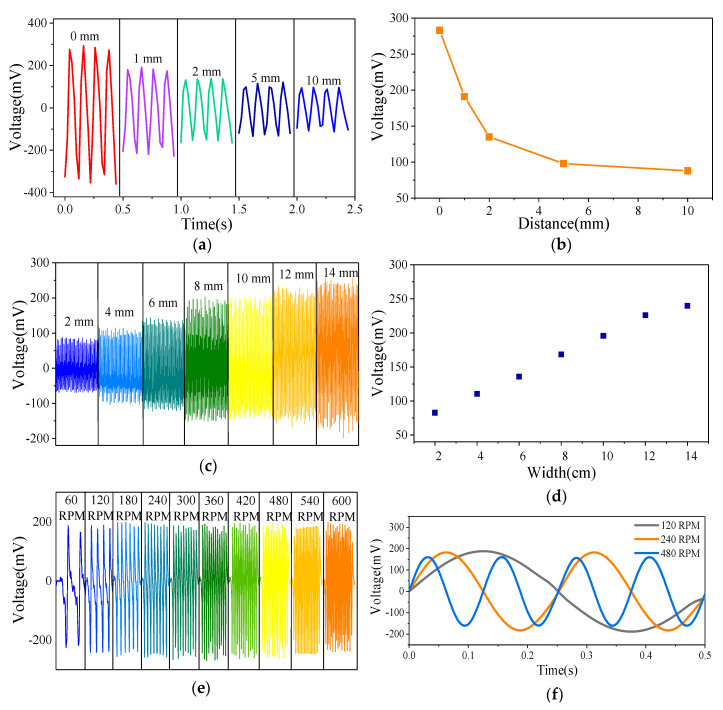
The influence of distance of two films on voltage waveform (**a**) and amplitude (**b**), the impact of PU film width on voltage waveform (**c**) and amplitude (**d**), output voltage at different rotational speed (**e**), sinusoidal fitting curve of voltage at 120 RPM, 240 RPM, 480 RPM (**f**).

To explore the effect of the distance between the films on the output voltage, the output voltage of the sensor with a film distance in the range of 0–10 mm is measured. Figure 3a,b show that with the other conditions unchanged, the output voltage of the SNTRS gradually decreases with the increase in the vertical distance between the PTFE and PU. When the distance is increased from 0 to 1 mm, the amplitude of the output voltage drops from 283 to 191 mV. When the distance is 10 mm, the output voltage drops to 88 mV. This is because the electrostatic induction becomes weaker as the spatial distance increases. In addition, although the amplitude of the output voltage changes, the output waveform is undisturbed by the increase in the distance. This result indicates that the proposed sensor can perform non-contact measurements. Controlling the distance between the films can help avoid friction between them in actual operation while ensuring a sufficiently strong signal output. The distance between the two films is set to 1 mm, and the bearing clearance is small. This ensures that the PU and PTFE films do not contact each other, and the amplitude of the output voltage is relatively high, which is conducive to the detection of the output signal.

To explore the impact of the film width on the output voltage, the distance between the PTFE and PU is fixed at 1 mm, and the width of the PTFE film is set to 10 mm. The width of the PU is increased from 2 mm, and then the output voltage of the sensor is measured. Figure 3c,d show that with the other conditions unchanged, the output voltage of the SNTRS gradually increases with the increase in the PU width. When the widths are 2 and 10 mm, the voltage values are 83 and 196 mV, respectively. In addition, when the width is increased to 14 mm, the voltage is 239 mV. This is because a PU film with a larger area can carry more charges. Output voltages with different amplitudes can be obtained by setting films with different widths, thus providing an experimental basis for the design of interdigital films.

To explore the impact of rotational speed on the output voltage, the distance between the PTFE and PU films is fixed at 1 mm, and the width of both the PTFE and PU films is set to 10 mm. The programmable motor is controlled to gradually increase the speed starting from 60 RPM. Figure 3e shows the change in the output voltage waveform with the increase in the motor rotational speed, with the other conditions unchanged. As the rotational speed increases, the frequency of the output voltage increases while the amplitude remains unchanged. This is because the measured voltage is an open circuit voltage, and no conduction current is formed [32]. Based on the output voltage law, a sine function is used to fit the output voltage of the SNTRS at 120, 240, and 480 RPM, respectively.

(1) $$y=y_{0}+Asin\left(\pi \frac{x-x_{c}}{w}\right)$$

The fitting parameters obtained are shown in Figure S1. Figure 3f shows the voltage function obtained after fitting. Figure 3f shows that at the three rotational speeds, the amplitudes of the output voltages are the same, and the output voltage frequencies double with the rotational speed, reflecting the accuracy of the SNTRS for rotational speed measurements.

The ability to maintain a high surface charge density for a long duration is a key factor for the long-term stable operation of non-contact sensors. To study the working stability of the SNTRS, after the PU film is pre-friction charged, the SNTRS is kept running for 4 h. As shown in Figure S1a, the output voltage gradually decreases and stabilizes within 4 h. The results indicate that, although the positive charges on the PU surface are partially lost during the rotation, a high charge level is still maintained for a long time, thus ensuring the normal operation of the SNTRS. Furthermore, a daily output voltage test is performed on the SNTRS. Figure S1b shows the result. In the first three days, the output voltage drops. Nevertheless, the voltage stabilizes after three days. Although the charge loss on the PU surface is inevitable, a certain amount of charge is retained for a long time and can then be detected. When the time is long enough, the amount of charge on PU and PTFE should be as much as that on PU and PTFE without pre-rubbing treatment. The experiment was conducted with PU and PTFE without pre-friction. The voltage was 0 mV at the beginning. However, during the rotation, the output voltage gradually increased, and a periodic voltage output of about 30 mV could be generated in 1 min. Based on this phenomenon, it is speculated that PU and PTFE films can be charged by friction with air during rotation, but the friction efficiency is lower than that of direct contact. Therefore, the charge on the PU and PTFE film of the SNTRS that has been rotating will not completely disappear, but always maintain a certain amount. Therefore, the charge on the PU and PTFE film of the SNTRS that has been rotating will not completely disappear, but always maintain a certain amount.

The experimental results prove that the amplitude of the output voltage is negatively correlated with the distance between the films and positively correlated with the film width. In addition, only the frequency of the output voltage affects the amplitude of the output voltage, not the motor rotation speed. Therefore, fixing the distance between the films and setting strip PUs of different widths at the same time can yield voltage peaks with different amplitudes in one rotation period.

From the experimental results shown in Figure 3, we find that the output voltage amplitude is positively correlated with the film width. Multiple PU films of different widths can be set on the stator to form an interdigital film, thereby increasing the sensing ability of the SNTRS. Considering the geometric size of the rotor, the maximum width of the PU film should not exceed 10 mm. Moreover, when the PU film width is less than 2 mm, the peak output voltage cannot be much higher than the signal noise. Therefore, the PU film width is set between 2 and 10 mm. When only one film is required, in order to increase the output voltage, a film with a width of 10 mm is selected. When two films are required, in order to facilitate the distinction, films with widths of 2 and 10 mm are chosen. When three films with different widths are used to form the interdigital sensitive layer, the minimum width of the PU film is set to 2 mm, and the maximum width is set to 10 mm, which ensures an optimum width range for the intermediate film. As shown in Figure 3d, the peak output voltages of the 2 and 10 mm PU films are approximately 70 and 190 mV, respectively. Evidently, the peak output voltage of the PU film with the middle width should be 130 mV to distinguish it from the low and high levels to the greatest extent. Hence, a width of 6 mm is chosen as the middle width. Considering a noise interference of ±20 mV, the peak voltage ranges of the 2, 6, and 10 mm PU films are 50–90, 110–150, and 170–210 mV, respectively. When processing the output signals, the analog quantity of the output voltage is converted to a digital quantity through A/D conversion. The calculation formula for the number of converter bits required is as follows.

(2) $$\frac{U_{max}}{\Delta U}\leq 2^{N}$$

where $U_{max}$ represents the maximum output voltage, which is 210 mV; ΔU is the minimum voltage that can be distinguished, which is 20 mV; and *N* is the number of bits of the A/D converter. From the calculation, interdigital films with widths of 2, 6, and 10 mm are set. In the subsequent signal processing, 4-bit A/D conversion is sufficient to meet the requirements, thus reducing the amount of calculation for signal processing.

**Figure 4:**
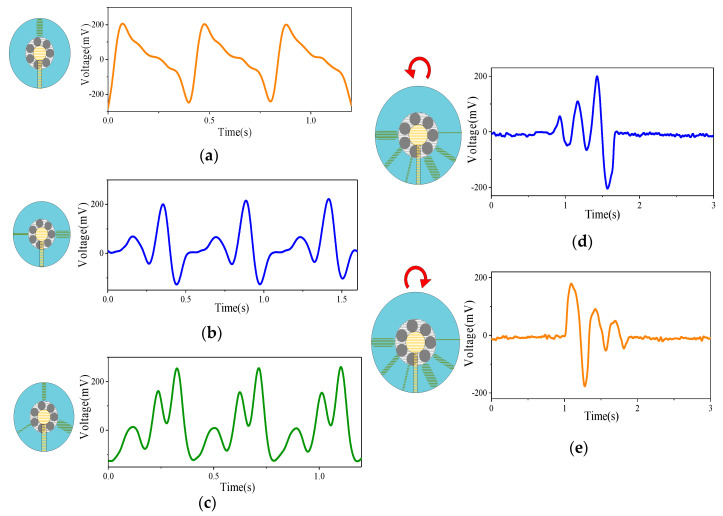
The output voltage of the sensor with 10 mm wide single interdigital PU film (**a**), 2 and 10 mm wide double interdigital PU film (**b**), 2, 6, and 10 mm wide three interdigit PU film (**c**). The output voltage of the sensor equipped with three interdigital PU film when rotated counterclockwise (**d**) and clockwise (**e**) by 120 degrees.

Figure 4a–e show the different voltage waveforms generated when different combinations of interdigital PU films are set on the rotor. When a single PU is set on the rotor, as shown in Figure 4a, there is only a single peak in each period of the output voltage. The time interval between two peaks represents the period of SNTRS rotation with the device. In Figure 4b, when PUs with widths of 2 and 10 mm sequentially sweep across the stator, two peaks with amplitudes of 71 and 193 mV are sequentially induced on the copper electrode of the stator. In Figure 4c, when three PUs with widths of 2, 6, and 10 mm are sequentially set on the stator, the output voltage has three small, medium, and large peaks in one period. The interdigital film helped enhance the control of the rotation process and increase the measurement accuracy. More importantly, it significantly improved the ability of the SNTRS to measure swing motion.

Figure 4d,e show the ability of the SNTRS with interdigital films to detect swing motion. The rotor swings 120° counterclockwise from the initial position, as shown in the figure, and then remains still. During the rotation, the stator sweeps the 2, 6, and 10 mm PUs in sequence. The output voltage starts from zero and returns to zero after the three small, medium, and large peaks. When the rotor rotates counterclockwise, the output voltage returns to zero after the occurrence of the three peaks (large, medium, and small) in sequence. Accordingly, the direction of the swing can be determined from the order in which the voltage peaks of different amplitudes appear. In addition, based on the number of peaks and the time interval between the peaks, the amplitude of the swing can be judged, such that the speed and acceleration of the swing process can be calculated.

Notably, compared to simply increasing the number of PU films, the interdigital film can increase the sampling frequency as well as help determine the direction of rotation from the output signals. As shown in Figure 4d, the output voltage peaks are in the order of small, medium, and large. Combined with the position of the interdigital film, it is evident that the motor rotates counterclockwise. Similarly, when the motor rotates clockwise, the peaks of the output voltage appear in the order of large, medium, and small. Additionally, the SNTRS with the interdigital film can feedback the real-time rotation position of the motor within a certain accuracy. In Figure 4d, after the appearance of the low peak and before the appearance of the medium peak, it can be determined that the stator is between the 2 and 6 mm PU. This is important information for controlling the motor to achieve complex rotation.

## 4. Application of SNTRS for Detecting Rotation and Swing Motion

**Figure 5:**
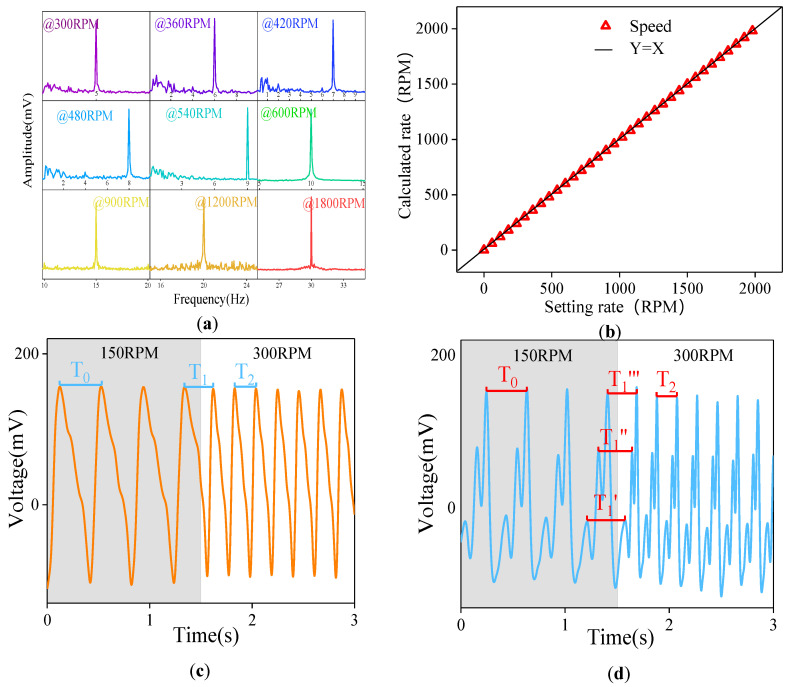
Amplitude–frequency curve at different rotational speeds (**a**), the speed measured by the sensor at 0–2000 RPM rotational speed (**b**). The transient output voltage of the sensor with 10 mm wide single interdigital PU film (**c**), 2, 6, and 10 mm wide three interdigital PU film (**d**) when the rotational speed is increased from 150 to 300 RPM.

The rotational speed measurement accuracy is the most important performance index of a rotational speed sensor. To explore the measurement accuracy of the SNTRS for the rotational speed, the rotational speed of the motor is increased from zero at 60 RPM intervals, and the voltage signals at each rotational speed are collected. A Fourier transform is performed on the collected signals, and a frequency–amplitude curve is plotted (Figure 5a). As shown in Figure 5a, when the motor rotation speed velocity is set to 300 RPM, the main frequency of the measured output voltage is 5 Hz. The following formula yields a speed of 300 RPM.

(3) $$n=\frac{60f}{p}$$

where *n* is the calculation speed (r/min), *f* is the detection frequency (Hz), and *p* is the number of pole pairs. This indicates that the output signals obtained from the SNTRS accurately reflect the actual rotational speed of the motor. The set rotational speed is increased at 60 RPM intervals, and the calculation speed is determined. Figure 5b shows the result. As shown, the set motor rotational speed and the calculation speed obtained through the SNTRS output voltage are the same, and the maximum error does not exceed 0.2%. The experimental results prove that the SNTRS can accurately measure rotational speeds below 2000 RPM.

For high-speed motors, the start–stop and acceleration/deceleration processes are called the transient processes of the motor. The transient processes have short durations and high control precision, which requires the rotational speed sensor to have a high measurement accuracy. In Figure 5c, the motor rotates at a speed of 150 RPM in the initial state, and the SNTRS with a single film is used to measure the motor rotational speed. The time interval T0 between two voltage peaks is 0.4 s, and the corresponding calculation speed is 150 RPM. At 1.5 s, the control system adjusts the motor rotational speed to 300 RPM, and the time interval T1 between the two voltage peaks adjacent to t = 1.5 s is 0.275 s. The transition process of the rotational speed is rapid. The time interval T2 between the first and second voltage peaks after 1.5 s is 0.2 s, and it continues to stabilize at 0.2 s thereafter. In fact, the SNTRS only collects the change process of the rotational speed in one period, which is insufficient for analyzing the change process of the motor rotational speed. For the SNTRS with only one thin film, when the transient process of the motor is short, the SNTRS only samples once in a rotation period, which results in a low sampling frequency, and the transient process is not fully reflected. The most direct method to improve the sampling frequency is to increase the number of PU films.

In Figure 5d, interdigital films with widths of 2, 6, and 10 mm are placed on the SNTRS rotor. Similar to the previous case, when t = 1.5 s, the control motor velocity is increased from 150 to 300 RPM. Before the rotational speed changes, the period of the SNTRS output voltage is T0 = 0.4 s, and the calculation speed is 150 RPM, which is equal to the set rotational speed. As the rotational speed varies, the time interval between two adjacent low peaks is measured to be T1′ = 0.36 s, the time interval between two medium peaks is T1″ = 0.32 s, and the time interval between two high peaks is T1″ = 0.26 s. These values are substituted into Equation (2) to obtain the corresponding calculation speeds: 166.7, 187.5, and 230.8 RPM, respectively. After the rotational speed is stabilized, we have T2 = 0.2 s, and the calculation speed is 300 RPM, which is the same as the set rotational speed. Based on the changes at the three calculation speeds, the transient process of the motor can be described more accurately. Therefore, the interdigital film can help improve the measurement accuracy of the SNTRS in the transient process of the motor.

**Figure 6:**
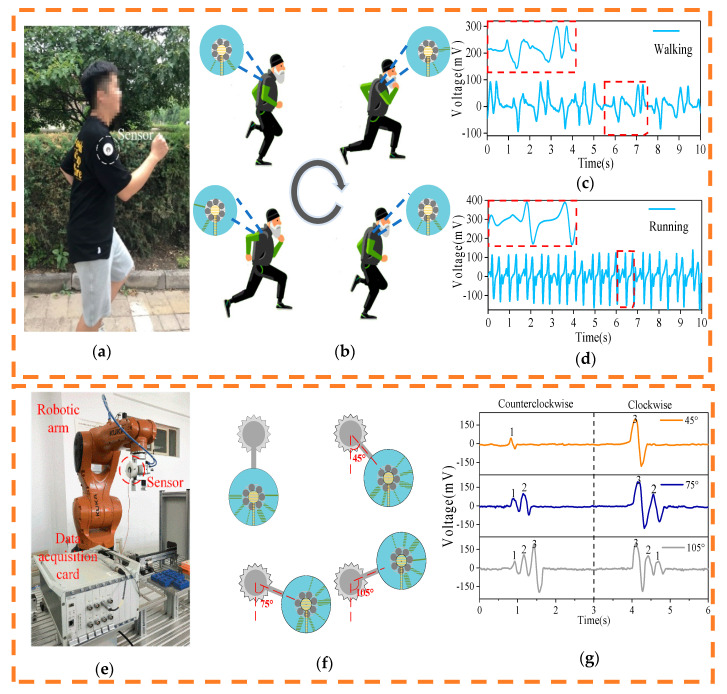
Photos (**a**) and schematic diagrams (**b**) of the sensor installed on the tester, and the output voltage of the sensor during walking (**c**) and running (**d**). The photo (**e**), working diagram (**f**), and output voltage (**g**) of the sensor installed on the robotic arm.

In addition to accurately detecting rotational motion, the SNTRS has excellent swing detection capabilities. As shown in Figure 6a, the SNTRS is fixed on a tester’s arm [33]. During walking and running, the rotor swings back and forth with the motion of the arm, whereas the stator remains perpendicular to the ground under the action of gravity. To distinguish the forward and backward swings of the arm as well as to reduce the complexity of the output signals as much as possible, a PU film with a width of 2 mm is set at the 5 o’clock position of the rotor, and a PU film with a width of 6 mm is set at the 7 o’clock position of the rotor. When the arm swings forward, the stator of the SNTRS rotates counterclockwise with the arm. As the tilt angle of the arm increases, the stator and the 6 mm PU film at the 7 o’clock position tend to approach each other, completely covering and moving away from each other until the arm swings to the highest point. During the ascending process, with the relative movement between the stator and the PU film, the output voltage of the SNTRS first increases and then decreases, exhibiting a voltage peak of 100 mV. When the arm swings forward to the highest point, it starts to move backwards. As before, the stator and the PU layer at the 7 o’clock position tend to approach each other, completely covering and moving away from each other. Subsequently, it returns to the initial position to complete the entire forward swing process. At the same time, a voltage peak of 100 mV appears again on the SNTRS. The appearance of the two 100 mV voltage peaks indicates that the upper arm completes a forward swing motion and returns to the starting position. The backward swing of the arm is similar to the forward swing. The only difference is that the 2 mm PU film set at the 5 o’clock position produces a voltage peak of approximately 60 mV. The forward and backward swings can be distinguished on the basis of the amplitude of the output voltage.

The tester walks and runs while wearing the SNTRS. Figure 6b,c show the output signals of the SNTRS. When walking, the period time for the arm to complete a forward swing–lower swing–back swing–lower swing is 2 s on average. When the tester runs, the same cycle time is only 0.8 s. Therefore, the movement state of the test subject can be distinguished by the time required to complete a swing arm cycle.

As shown in Figure 6d,e, the SNTRS is installed on a programmable swing arm. The controller drives the motor, and the swing arm swings about the axis at any angle. The control motor rotates 45° counterclockwise from the initial position and then remains still. The SNTRS outputs a voltage peak with a peak value of 50 mV. On the other hand, when the control motor rotates 45° clockwise from the initial position and then remains still, the peak value outputted from the SNTRS is measured to be 200 mV. Similarly, when the motor is controlled to swing 75° and 105°, the output voltage of the SNTRS is recorded as shown in Figure 6e. The results show that the SNTRS can determine the swing direction and approximate swing angle from the number of output voltage peaks, the size of the voltage peaks, and the order in which the peaks with different peak values appear. In the actual operation, the working process of the swing arm is to distribute the workpiece on the assembly line to the shelves with different heights. When the swing arm puts the workpiece on the lower shelf, the rotation angle of SNTRS is in the range of 30–60 degrees. The rotation angle of the middle shelf is about 60–90 degrees, and the one of high shelf is more than 90 degrees. According to the changes of the voltage, whether the actual working process of the manipulator is consistent with the set process can be judged. In addition, SNTRS can adapt to different heights by adjusting the position of the PU film.

The above test proves that the SNTRS exhibits good performance for rotational speed measurement and swing sensing, making it applicable to motion sensing and control of robots. There is no doubt that the accuracy of measurement can be improved by increasing the number of PU sensitive films. In addition, based on its self-powered and non-contact characteristics, the SNTRS can work for a long time without maintenance and is cost-effective. The sensor has broad application prospects in fields such as wind power equipment, offshore equipment, and IoT.

## 5. Conclusions

In summary, a self-powered non-contact sensor based on the triboelectric effect was developed to detect rotation and swing motion. The SNTRS connected using a bearing works when it is fixed on a rotating structure, without requiring any fixing mechanism on the ground or operating table. The SNTRS comprises a PU film and a PTFE film, utilized for perceiving the rotation. The scanning electron microscopy results showed that the surface area of the PU sensitive film can be effectively increased through wet etching with sulfuric acid. The electric field distribution of the sensor was analyzed by finite element analysis. The relationship between the film distance and the output voltage amplitude was determined experimentally. The results showed that the non-contact sensing has fine output characteristics. The test and analysis results showed that the output voltage amplitude increases with the increase in the film width. Three-finger interdigital films with widths of 2, 6, and 10 mm were designed. A 4-digit A/D converter was sufficient to meet the sensing requirements in the subsequent signal processing. The interdigital film enables the sensor to distinguish the direction of rotation and swing, obtain real-time information of the rotation and swing, and sense transient processes such as rotational speed changes. In the speed range of 0–2000 RPM, the maximum error in the rotational speed measurement was only 0.2%. As part of an experiment, the sensor was installed on the arm of a tester. It took 2 and 0.8 s to finish a complete swing arm process during walking and running, respectively. This indicates that the sensor can be used to judge the motion state of the human body. Moreover, the tests conducted on the robotic arm showed that the sensor can recognize the swing direction and swing angle. Although the proposed sensor has a good sensing ability for rotation and swing, it cannot outperform existing encoders and other conventional sensors in terms of accuracy. Further improvements should be made to the structure and materials used in the future.

## Supplementary Materials

The following are available online at https://www.mdpi.com/1424-8220/20/17/4947/s1, Figure S1: Attenuation of voltage amplitude in 3 h (a) and 7 days (b), Table S1: The parameters of the fitting curve based on sine function at 120, 240, and 480 RPM.
