# Supplementary material for: Self-Powered Non-Contact Triboelectric Rotation Sensor with Interdigitated Film
Source: Sensors (Basel). 2020 Sep 1;20(17):4947. doi: 10.3390/s20174947 (PMC7506670; doi:10.3390/s20174947)
Supplement: Supplementary file 1 [file sensors-20-04947-s001.pdf]

Table S1. The parameters of the fitting curve based on sine function at 120, 240 and 480 RPM

Speed [RPM]	y_0	$\sigma(y_0)$	xc	σ(xc)	w	σ(w)	A	σ(A)
120	0.76822	1.22668	0.00112	0.00135	0.25253	0.00115	189.33237	1.81047
240	1.94304	1.04477	-2.29E-04	5.77E-04	0.12558	2.46E-04	189.65583	1.51199
480	1.25661	1.30176	1.12E-05	3.61E-04	0.06259	7.68E-05	188.51751	1.87564

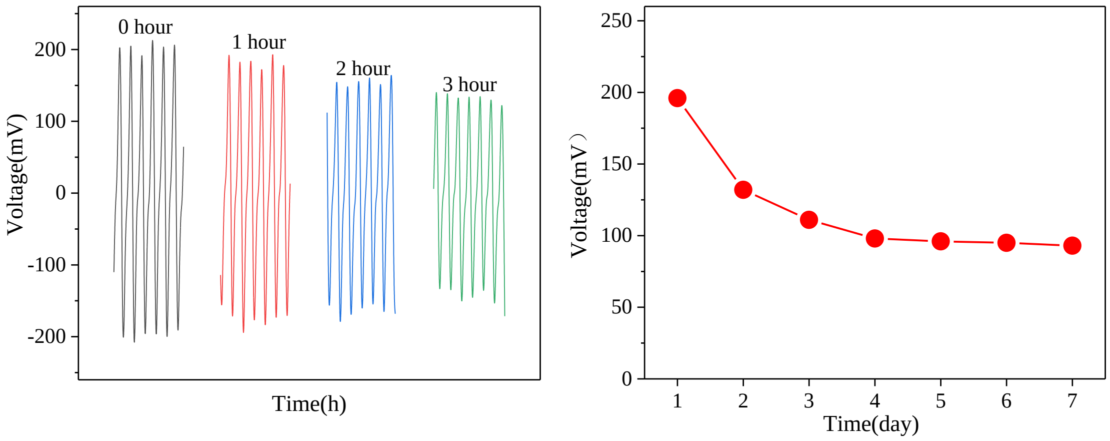

Figure S1. Attenuation of voltage amplitude in 3 hours (a) and 7 days (b)
